# The combined detection of aspiration biopsy, computed tomography and *BRAF^V600E^* gene has high diagnostic value for papillary thyroid carcinoma

**DOI:** 10.20945/2359-4292-2024-0182

**Published:** 2025-09-24

**Authors:** Peizhi Fan, Zhaoyi Wu, Zhecheng Li, Huiting Ouyang, Jianing Yi, Jie Yu

**Affiliations:** 1 Department of Thyroid and Breast Surgery, Hunan Provincial People’s Hospital, The First Affiliated Hospital of Hunan Normal University, Changsha, China; 2 Department of General Surgery, Xiangya Hospital, Central South University, Changsha, China

**Keywords:** Ultrasound-guided fine-needle aspiration biopsy, *BRAF^V600E^* gene test, papillary thyroid carcinoma, computed tomography, pathological tissue type

## Abstract

**Objective:**

This study investigated the clinical value of ultrasound-guided fine-needle aspiration biopsy (US-FNAB), computed tomography (CT) and *BRAF^V600E^* combination for papillary thyroid carcinoma (PTC) diagnosis.

**Subjects and methods:**

A total of 300 patients with thyroid nodules were assigned to the PTC group (n = 184) and the nodular goiter (NG) group (n = 116). The positive detection rates of US-FNAB, CT and *BRAF^V600E^* gene mutation and their relationship with tumor number, tumor diameter, lymphatic metastasis, capsule invasion and tumor-node-metastasis (TNM) staging were analyzed, with their diagnostic value for PTC analyzed by the receiver operating characteristic (ROC) curve. The area under multiple ROC curves (AUCs) were compared using MEDCALC software.

**Results:**

The positive detection rates of US-FNAB, CT and *BRAF^V600E^* gene mutation were 78.80%, 72.28% and 83.15% in the PTC group, and 30.17%, 27.59% and 9.48% in the NG group, while the negative detection rates were 21.20%, 27.72% and 16.85% in the PTC group, and 69.82%, 72.41% and 90.52% in the NG group. Positive US-FNAB and *BRAF^V600E^* gene mutation in PTC patients related to TNM staging. Positive CT and *BRAF^V600E^* gene mutation linked to lymphatic metastasis. US-FNAB (AUC: 0.743, sensitivity: 78.80%, specificity: 69.83%), CT (AUC: 0.723, sensitivity: 77.28%, specificity: 72.41%) and *BRAF^V600E^* (AUC: 0.868, sensitivity: 83.15%, specificity: 90.52%) gene detections helped PTC diagnosis, with their combined diagnostic value (AUC: 0.938, sensitivity: 78.26%, specificity: 96.55%) surpassing that of them alone.

**Conclusion:**

US-FNAB, CT and *BRAF^V600E^* gene tests helped PTC diagnosis, and their combined detection had higher diagnostic value for PTC than their single detection.

## INTRODUCTION

Thyroid cancer stands out as a prevailing malignant tumor among head and neck tumors and in the endocrine system in China, whose global incidence has reportedly emerged as a significant upward trend in the past 30 years (^1^). In 2022, thyroid cancer ranked third in incidence among all malignant tumors in China, and a total of approximately 466,100 new cases of thyroid cancer were reported, accounting for about 9.7% of all diagnosed malignancies in that year (^2^). Currently, thyroid cancer is mainly clinically classified based on its pathology, among which papillary thyroid carcinoma (PTC) accounts for up to 90%, and is the thyroid cancer with the highest incidence rate in the clinic (^3^). Moreover, PTC is a type of differentiated thyroid cancer mainly stemming from the follicular epithelial cells of the thyroid gland and having a lower degree of malignancy and a slower development relative to undifferentiated cancer, with a 5-year survival rate of > 90%, which has been considered as the “mildest” cancer in terms of biological behaviors (^4^-^6^). Importantly, clinical practice research has manifested that despite a good prognosis, PTC has no specific clinical manifestations and no obvious abnormalities in laboratory thyroid tests, and has been recognized with a high rate of occurrence of cervical lymph node metastasis in the central region, which has some bearing on the choice of treatment plan, specific death, prognosis and postoperative recurrence of patients with PTC (^7^,^8^).

Computed tomography (CT), ultrasonography, positron emission tomography/CT and magnetic resonance all have certain clinical value in the diagnosis and evaluation of PTC patients’ conditions, among which ultrasonography possesses advantages such as low cost, low radiation, convenience and high speed, so it has become the main method for diagnosing and evaluating PTC (^9^-^12^). Nevertheless, a single ultrasound examination has an incomprehensive evaluation for PTC, and the clinical diagnostic criteria for ultrasound thyroid nodule TI-RADS grading are still controversial. Therefore, it is of great significance to figure out an effective auxiliary examination to enhance the specificity and sensitivity of the diagnosis for PTC, and to accomplish the accurate diagnosis and complete evaluation of PTC.

Domestic and foreign scholars have long been attempting to use ultrasound-guided fine-needle aspiration biopsy (US-FNAB) to assist in the diagnosis and evaluation of PTC, and identified that US-FNAB has the capacity to make a pathological cytological diagnosis and determine benignity or malignancy of the nodules (^13^,^14^). However, it has been implied that the limitation of ultrasound examination in US-FNAB inevitably leads to incomplete assessment of cervical lymph nodes and a certain false-negative rate (^15^). Furthermore, US-FNAB has certain limitations in assessing the classification of thyroid tumors, necessitating supplementary molecular detection or other ancillary investigations (^16^). Hence, it remains highly controversial whether PTC patients clinically need prophylactic neck lymph node dissection.

CT is also a common non-invasive examination method in the diagnosis and treatment of PTC. In comparison to ultrasonography, CT offers superior advantages in observing central lymph node metastasis, invasion of neighboring tissues and organs, and coarse calcification (^17^-^19^). CT can provide detailed information on thyroid anatomical location for clinical medical workers, especially in the relationship between lymph node location and anatomic nodal locations (^20^). However, as the size of the nodule decreases, the detection rate of PTC by CT gradually declines (^21^).

It is worth noting that the researchers have gradually changed their focus to basic research of PTC in recent years, with the aim to find PTC-related genes or small molecules, which have been suggested by the American Thyroid Association Guide to have the potential to assist in the diagnosis of PTC (^22^). At present, BRAF mutations stand out as one of the most representative molecular events in the occurrence of PTC, with an incidence of 30%-80%, comprising an exchange of valine for *BRAF^V600E^* (^23^,^24^). Notably, Chen and cols. have revealed that *BRAF^V600E^* mutations are closely associated with local recurrence, disease-specific death and aggressiveness in PTC (^25^). Although most studies have shown that the *BRAF^V600E^* mutation is significantly linked with PTC, other researchers have found that false-positive results still exist in *BRAF^V600E^* mutation detection (^26^).

At present, there are few clinical reports about US-FNAB, CT and *BRAF^V600E^* gene tests. To further clarify the diagnostic value of US-FNAB, CT combined with *BRAF^V600E^* gene tests for PTC, increase the diagnostic yield, strengthen the evaluation of the condition, and provide the scientific and reasonable basis for formulating the patients’ subsequent treatment and follow-up program, we investigated the clinical value of combining US-FNAB, CT and *BRAF^V600E^* gene detections in the diagnosis and treatment of PTC.

## SUBJECTS AND METHODS

### Ethics statement

This study was reviewed and approved by the Academic Ethics Committee of Hunan Provincial People’s Hospital, The First Affiliated Hospital of Hunan Normal University (2019-1223), and was in line with the Declaration of Helsinki and the Enhancing the Quality and Transparency Of health Research network guidelines. All subjects were informed of the purpose of the study and signed the informed consent forms.

### Sample size estimation

**Supplementary figure 1:**
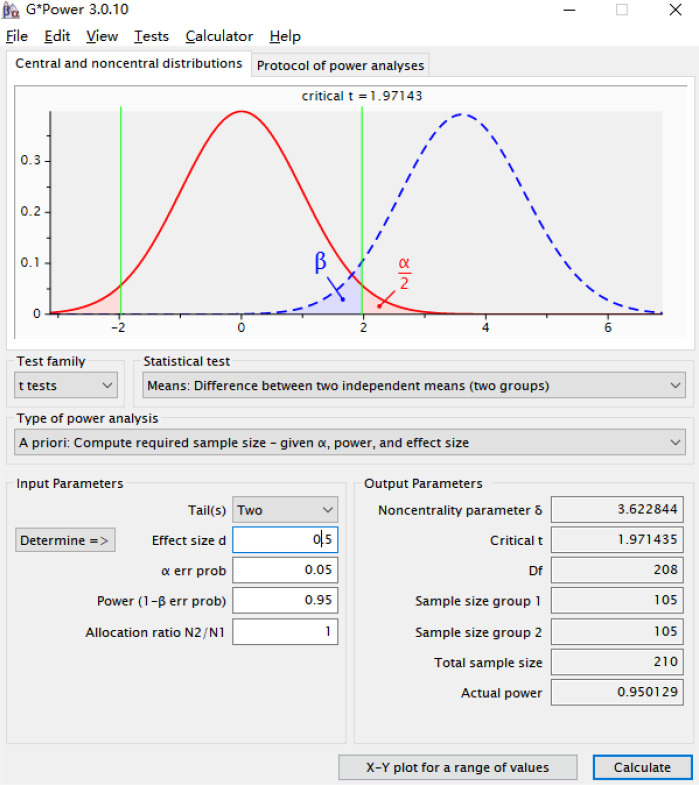
Sample size estimation.

In this study, sample size estimation was conducted using the G*Power 3.0.10 software (Heinrich-Heine-Universität Düsseldorf, Germany) (**Supplementary Figure 1**). The testing method selected was the independent-sample *t*-test, with the following setting parameters: α = 0.05, β = 0.95, effect size = 0.5, and *P* was obtained from a two-tailed test. The estimated results illustrated that the minimum required sample size was 210 patients.

### Research subjects

This study included 372 patients with thyroid nodules who visited the outpatient department of Hunan Provincial People’s Hospital, The First Affiliated Hospital of Hunan Normal University from January 2020 to March 2023. According to the inclusion criteria, 346 patients were included, among which 46 patients were excluded as per the exclusion criteria. Finally, 300 patients were selected as the study subjects, among which 184 patients who conformed to the diagnostic criteria of PTC in the American Guidelines for the Clinical Diagnosis and Treatment of Thyroid Nodules and Differentiated Thyroid Cancer, and were diagnosed as PTC by postoperative histopathological examination were included as the PTC group, and 116 patients who were definitely diagnosed as nodular goiter (NG) of benign lesions were enrolled as the NG group.

### Inclusion and exclusion criteria

The inclusion criteria were as below: (^1^) underwent US-FNAB, CT and *BRAF^V600E^* gene detections before surgery and underwent surgical resection of thyroid nodules; (^2^) first visit to the clinic; (^3^) 18 < age < 80 years; (^4^) had previously no related treatment including radiofrequency ablation or thyroidectomy; (^5^) had no history of exposure to radioactive substances in the neck; (^6^) with complete data.

The exclusion criteria were as follows: (^1^) a history of related thyroid function abnormalities such as Hashimoto’s thyroiditis, hyperthyroidism with the thyroid function turned normal following treatment; (^2^) a familial history of PTC (with ≥ 3 immediate family members having highly-differentiated thyroid cancer); (^3^) controversial and undefined results of related examinations and test (^4^) a history of tumors in other sites (^5^) complication with multiorgan failure; (^6^) pregnant and lactating women (^7^) postoperative pathology results showing thyroid malignant tumors that were not papillary carcinoma.

PTC pathological diagnostic criteria were as follows: PTC patients were diagnosed by clinical pathological examination, as evidenced by the papillary structure of different sizes, solid nest-like focal area, glassy or transparent cell nuclei within pseudo-inclusion bodies and nuclear grooves, and balanced distribution of fine-grained chromatin; besides, interstitial fibrous tissues displayed hyperplasia along with obvious hyalinization under the microscope, and the mass was hard, with gray-brown surface and grayish-white cut surface.

### Data collection

Clinical baseline data including age, sex, body mass index (BMI) and puncture site of all study subjects, as well as the number of tumors, tumor diameter, capsule invasion, lymphatic metastasis, tumor-node-metastasis (TNM) staging and the results of pathologic examination from US-FNAB, CT and *BRAF^V600E^* genetic tests in PTC patients were collected.

### US-FNAB test

Subjects were kept in the supine position, with the neck elevated to fully reveal the puncture site. The nodule to be examined was punctured under the localization using a Color Doppler Ultrasonography diagnostic instrument (VIVID 5, Massachusetts, GE, USA). The orientation of the puncture needle was changed under negative pressure conditions, and the specimen was absorbed utilizing the rapid fan-shaped multi-point puncture method, with the specimen repeatedly absorbed ≥ 5 times. After the elimination of the negative pressure and removal of the needle, the puncture point was pressed using a sterile cotton ball for 5 min. A part of the specimen was quickly coated and fixed for cytology, while the other part of the tissue was injected into the pathology specimen bottle and stored at 2-8 °C for genetic test. US-FNAB operations were performed by the same senior ultrasound physician using a unified puncture method (rapid fan-shaped multi-point puncture method). The US-FNAB outcomes were interpreted by a senior pathologist according to the Bethesda diagnostic system developed by the Thyroid Association of the National Cancer Institute of America, and were reviewed blindly by two senior pathologists. In this study, the positive results of US-FNAB were defined as follows: the results of the puncture report suggested that it was suspected to be PTC or consistent with the manifestations of papillary carcinoma cells (pieces of hyperplastic thyroid follicular epithelial cells could be seen, with crowded cell arrangement, and visible multinucleated giant cells, intranuclear pseudoinclusions and nuclear grooves). It was considered negative if the aforementioned description was not observed.

### CT detection

All metal material items on the patient were removed, and the patient was then kept in a supine position, with the neck extended back as far as possible to fully expose the thyroid gland in the anterior region of the neck. Before the examination, the patients were instructed to hold his breath and not swallow. A 256-slice spiral CT scanner (Brilliance ICT, Philips, Amsterdam, Netherlands) was applied with a scanning pitch of 1.00, a layer thickness of 5.00 mm, and a layer spacing of 5.00 mm. The scanning range was from the horizontal plane of the mandible to the sternoclavicular joint. Elder patients were supposed to have a limited extension to avoid obstruction of vertebral artery blood flow. After a thyroid CT scan examination, patients were injected with a contrast agent by nurses to perform an enhanced CT scan of the thyroid gland. Ioversol was used as a contrast agent, which was injected into the elbow vein at a flow rate of 2.00-3.00 mL/s using a high-pressure syringe. CT images were used for diagnosis by two experienced physicians using a double-blind method. In case of disagreement of the diagnosis results, they needed to discuss to reach a unanimous conclusion.

### *BRAF^V600E^* genetic test

On the day of specimen collection, DNA was extracted from histopathological specimens collected through the US-FNAB test, and *BRAF^V600E^* gene mutation was detected by real-time quantitative polymerase chain reaction (RT-qPCR). The detection process was as below: DNA was extracted from the histopathological specimens utilizing a DNA extraction kit (N902, Vazyme Biotech, Nanjing, Jiangsu, China) and placed in 50 µL of buffer ATE (included in the kit), followed by the DNA absorbance measurement using a micro UV spectrophotometer (SMA4000, Merrill Lynch Hengtong Instruments, Beijing, China). Subsequently, the DNA concentration was diluted to 2-3 ng/µL with distilled water. The mixture was prepared according to the addition standard of 5 µL DNA sample solution and 0.4 µL of Taq enzyme for every 35 µL of reactive mix, and each PCR reaction tube was added with 35 µL of reactive mix (the amount of DNA in a single PCR reaction tube ranged from 10 to 15 g). After centrifugation, the reaction tube was placed into a real-time PCR instrument (4376373, Applied Biosystems, Foster City, CA, USA) for determination, with the conditions set as 95 °C, 5 min, 15 cycles of 95 °C, 25 s, 64 °C, 20 s and 72 °C, 20 s, and 31 cycles of 93 °C, 25 s, 60 °C, 35 s and 72 °C, 20 s. The FAM and HEX signals were collected at 60 °C, with real-time PCR performed and the document preserved. The results of gene mutation were determined by the cycle threshold (Ct) value of the FAM signal. If the Ct value of the FAM signal is less than 28, the sample is considered negative (or below the detection limit of the kit). Conversely, if the Ct value of the FAM signal is 28 or higher, the sample is deemed positive. In this study, *BRAF^V600E^* gene mutation was viewed as positive results of the *BRAF^V600E^* genetic test, and the remaining outcomes were determined as negative results.

### Statistical analysis

Statistical analyses and graphing were conducted on data using SPSS 21.0 statistical software (IBM Corp., Armonk, NY, USA) and MedCalc 19.0 software (MedCalc Software, Ostend, Belgium). Normal distribution was tested using the Shapiro-Wilk test. Measurement data in line with normal distribution were represented in the form of mean ± standard deviation. Comparisons between groups were implemented using an independent sample *t*-test. Counting data were expressed as the number of cases, and comparisons between groups were performed by the Chi-square test. The receiver operating characteristic (ROC) curve was plotted to evaluate the diagnostic value of US-FNAB test, CT, *BRAF^V600E^* genetic test, and the combination of the three. Comparisons of multiple area under multiple ROC curves (AUCs) were performed using the DeLong test in MedCalc software. The test level was set as a = 0.05. *P* was a two-sided test, and *P* < 0.05 was regarded as statistically significant.

## RESULTS

### Baseline data characteristics

We compared and analyzed the clinical baseline data of patients between the PTC group and the NG group. There were no statistically significant differences in clinical baseline data, including age, BMI, sex, puncture site and nodule number, between the two groups (**Table 1**) (all *P* > 0.05).

**Table 1:** General information of the enrolled population

	PTC group (n = 184)	NG group (n = 116)	*P* value
Age (years)	43.02 ± 11.32	42.96 ± 10.63	0.512
BMI (kg/m^2^)	21.20 ± 2.23	21.07 ± 3.19	0.211
Sex (male)	30 (16.30%)	28 (24.14%)	0.101
Puncture site			
Left side	70 (38.04%)	41 (35.34%)	0.892
Right side	69 (37.50%)	45 (38.79%)	
Bilateral	45 (24.46%)	30 (25.86%)	
Number of nodules			
Single	114 (61.96%)	66 (56.90%)	0.399
Multiple	70 (38.04%)	50 (43.10%)	

Note: BMI, body mass index. The count data were expressed by the number of cases and percentages, and the Chi-square test was used between groups. Measurement data were expressed as mean ± standard deviation, and independent sample *t* test was used for inter-group comparisons.

### The positive detection rates of US-FNAB, CT and *BRAF^V600E^* gene mutation were higher in PTC patients than in patients with benign thyroid nodules

We further compared the results of US-FNAB, CT and *BRAF^V600E^* gene detections between the two groups. As shown in **Table 2**, in the PTC group, the numbers of patients showing positive results for US-FNAB, CT and *BRAF^V600E^* gene tests were 145 (78.80%), 133 (72.28%) and 153 (83.15%), respectively, whereas those of patients exhibiting negative results for US-FNAB, CT and *BRAF^V600E^* gene detections were 39 (21.20%), 51 (27.72%) and 31 (16.85%), respectively. However, in the NG group, there were separately 35 (30.17%), 32 (27.59%) and 11 (9.48%) patients who showed positive results for US-FNAB, CT, and *BRAF^V600E^* gene detections, respectively, and 81 (69.82%), 84 (72.41%) and 105 (90.52%) patients who exhibited negative results for US-FNAB, CT, and *BRAF^V600E^* gene tests, respectively. The PTC group had much higher positive rates for the US-FNAB, CT, and *BRAF^V600E^* gene tests than the NG group (all *P* < 0.01). These results hinted that the positive detection rates of US-FNAB, CT and *BRAF^V600E^* gene mutation in PTC patients were higher than those in patients with benign thyroid nodules.

**Table 2:** The positive rates of US-FNAB, CT and *BRAF^V600E^* gene mutation were high in PTC patients

		PTC group (n = 184)	NG group (n = 116)	*P* value
US-FNAB detection	Positive	145 (78.80%)	35 (30.17%)	<0.001
	Negative	39 (21.20%)	81 (69.82%)	
CT detection	Positive	133 (72.28%)	32 (27.59%)	<0.001
	Negative	51 (27.72%)	84 (72.41%)	
*BRAF^V600E^* gene detection	Positive	153 (83.15%)	11 (9.48%)	<0.001
	Negative	31 (16.85%)	105 (90.52%)	

Note: The count data were expressed by the number of cases and percentages, and the Chi-square test was used between groups.

### Relationship between positive US-FNAB, CT and *BRAF^V600E^* gene mutation and PTC clinicopathologic features in PTC patients

Subsequently, we analyzed the relationship of positive US-FNAB, CT and *BRAF^V600E^* gene mutation with PTC clinicopathologic features, and found that (**Table 3**) the difference was statistically significant in terms of positive detection rate of US-FNAB between PTC patients at different TNM stages (*P* < 0.05), wherein PTC patients at TNM stage I had a US-FNAB positive detection rate of 73.58%, and those at stages II-III had an 85.90% US-FNAB positive detection rate. Nevertheless, there was no significant difference in US-FNAB positive detection rate between PTC patients in terms of tumor number, tumor diameter, capsule invasion or lymphatic metastasis (all *P* > 0.05). The positive detection rate of *BRAF^V600E^* gene mutation in PTC patients was statistically different in lymphatic metastasis and TNM staging (all *P* < 0.05). The positive detection rate of *BRAF^V600E^* gene mutation in PTC patients without lymphatic metastasis was 77.57%, whereas in those with lymphatic metastasis, it was 90.91%. The positive detection rate of the *BRAF^V600E^* gene mutation in patients with PTC at TNM stage I was 76.42%, while the rate for those at TNM stages II-III was 92.31%. However, no significant disparity was found in the positive detection rate of *BRAF^V600E^* gene mutation in tumor number, tumor diameter or capsule invasion (all *P* > 0.05). Furthermore, the positive detection rate of CT in patients with PTC exhibited a statistically significant difference in cases of lymphatic metastasis (*P* < 0.05). PTC patients without lymphatic metastasis had a CT-positive detection rate of 66.36%, while those with lymphatic metastasis had a CT-positive detection rate of 80.52%; yet, the US-FNAB positive detection rate in PTC patients did not differ significantly in tumor number, tumor diameter, capsule inv asion, or TNM staging (all *P* > 0.05). These findings implied that the results of US-FNAB were related to TNM staging, the results of *BRAF^V600E^* gene mutation were related to lymphatic metastasis and TNM staging, and the results of CT were linked to lymphatic metastasis in PTC patients.

**Table 3:** The relationship between US-FNAB, *BRAF^V600E^* gene mutation and CT and PTC clinicopathological features

	US-FNAB		*P* value	*BRAF^V600E^* gene mutation		*P* value	CT		*P* value
	Positive (n = 145)	Negative (n = 39)		Positive (n = 153)	Negative (n = 31)		Positive (n = 133)	Negative (n = 51)	
Number of tumors									
Single (n = 114)	93 (81.58)	21 (18.42)	0.268	95 (83.33)	19 (16.67)	0.933	84 (73.68)	30 (26.32)	0.614
Multiple (n = 70)	52 (74.29)	18 (25.71)		58 (82.86)	12 (17.14)		49 (70.00)	21 (30.00)	
Tumor diameter									
<0.5 cm (n = 111)	89 (80.18)	22 (19.82)	0.585	92 (82.88)	19 (17.12)	0.904	78 (70.27)	33 (29.73)	0.503
≥0.5 cm (n = 73)	56 (76.71)	17 (23.29)		61 (83.56)	12 (16.44)		55 (75.34)	18 (24.66)	
Capsule invasion									
No (n = 105)	81 (77.14)	24 (22.86)	0.587	84 (80.00)	21 (20.00)	0.234	72 (68.57)	33 (31.43)	0.244
Yes (n = 79)	64 (81.01)	15 (18.99)		69 (87.34)	10 (12.66)		61 (77.22)	18 (22.78)	
Lymphatic metastasis									
No (n = 107)	80 (74.77)	27 (25.23)	0.144	83 (77.57)	24 (22.43)	0.018	71 (66.36)	36 (33.64)	0.045
Yes (n = 77)	65 (84.42)	12 (15.58)		70 (90.91)	7 (9.09)		62 (80.52)	15 (19.48)	
TNM staging									
Stage I (n = 106)	78 (73.58)	28 (26.42)	0.047	81 (76.42)	25 (23.58)	0.005	72 (67.92)	34 (32.08)	0.136
Stages II-III (n = 78)	67 (85.90)	11 (14.10)		72 (92.31)	6 (7.69)		61 (78.21)	17 (21.79)	

Note: Count data were expressed as number of cases and percentages, and the Chi-square test was used between groups.

### Combining US-FNAB, CT and *BRAF^V600E^* gene tests had high diagnostic value for PTC

**Figure 1:**
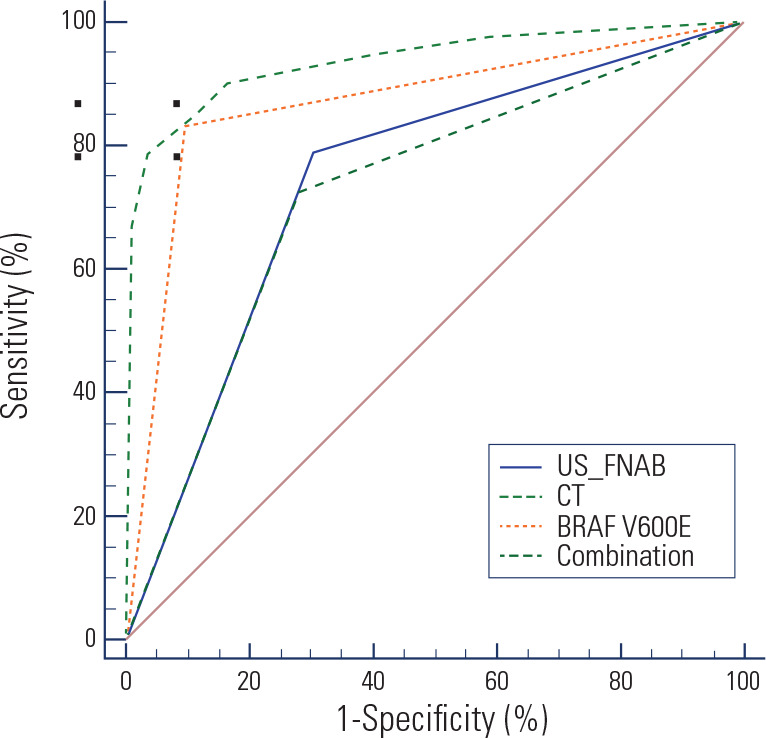
Diagnostic efficacy of the combination of US-FNAB, CT and *BRAF^V600E^* gene tests for PTC. ROC curves were plotted to analyze the diagnostic efficacy of US-FNAB detection, CT detection and *BRAF^V600E^* gene detection and the combination of the three for PTC.

To further probe the clinical diagnostic value of CT, US-FNAB combined with *BRAF^V600E^* gene tests for PTC patients, we analyzed the results of ROC curves (**Table 4, Figure 1**). AUCs of US-FNAB, CT and *BRAF^V600E^* gene detections for diagnosing PTC were 0.743, 0.723, and 0.868, respectively, with the sensitivities of 78.80%, 77.28% and 83.15%, and the specificities of 69.83%, 72.41% and 90.52%. This suggested that US-FNAB test, CT test and *BRAF^V600E^* gene test all had certain diagnostic efficacy for PTC. In addition, the AUC of the combined detection of the three for PTC diagnosis was 0.938 (78.26% sensitivity and 96.55% specificity), which was higher than that of their single detection (all *P* < 0.001). Overall, it could be concluded that the detections of US-FNAB, CT and *BRAF^V600E^* gene all had high diagnostic value for PTC, with their combined detection showing higher diagnostic value than them individually.

**Table 4:** Diagnostic efficacy of US-FNAB, CT and *BRAF^V600E^* genetic tests for PTC

Item	Sensitivity	Specificity	AUC	P	95% CI
US-FNAB	78.80	69.83	0.743	<0.001	0.690-0.792
CT	77.28	72.41	0.723	<0.001	0.669-0.773
*BRAF^V600E^*	83.15	90.52	0.868	<0.001	0.825-0.904
Combination	78.26	96.55	0.938	<0.001	0.905-0.963
US-FNAB~combination	P < 0.001				
CT~combination	P < 0.001				
*BRAF^V600E^*~combination	P < 0.001				

Note: AUC, area under the receiver operating characteristic curve. Multiple AUC comparisons were performed using the Delong test in MEDCALC software. *P* < 0.05 was considered to demonstrate statistically significant differences.

## DISCUSSION

The prevalence of PTC is around 80%-90% of all primary thyroid cancers, uniquely featured by predominate occurrence in females relative to multiple other cancers (^27^). Due to the rising incidence and youth-oriented tendency of thyroid carcinoma, early intervention and regular medical examinations are necessary (^28^). Lv and cols. have found that *BRAF^V600E^* gene mutation is linked to uneven edges of nodules, age ≤ 46.5 years old and abnormal lymph nodes in the neck in PTC patients, showing some guiding importance for clinical diagnosis, treatment and prognosis (^29^). CT, a standard clinical imaging technique, can illustrate intricate and objective anatomical details and may offer numerous prognostic factors for PTC patients (^30^,^31^). Besides, there is growing evidence showing that US-FNAB has become a quick, reliable and cost-effective diagnostic procedure in the evaluation of thyroid nodules in the past few decades (^32^-^34^). Our findings highlighted that US-FNAB, CT and *BRAF^V600E^* gene detection results were involved in the clinicopathologic features of PTC patients to some extent, and aided in the diagnosis of PTC occurrence, with their combination showing high diagnostic value for PTC.

US-FNAB is a secure, quick and accurate technique that can be conducted without anesthesia in an outpatient setting, and is widely regarded as the “gold standard” for preoperative assessment of the benign or malignant characteristics of thyroid nodules (^22^,^35^). However, the outcomes of US-FNAB are largely contingent upon the expertise and technical proficiency of the puncturing physician, while CT serves as a valuable complement to sonography, compensating for its limitations (^36^,^37^). Additionally, due to being limited by ultrasound examination, there is a certain false negative rate in US-FNAB (^15^). Other research indicates that *BRAF^V600E^* analysis enhances the diagnostic precision of fine needle aspiration and declines the false negative rate (^38^). In this regard, we assumed that integrating US-FNAB, CT and *BRAF^V600E^* gene detections was more advantageous for the diagnosis of PTC. PTC and NG represent the predominant malignant and benign thyroid nodules in incidental thyroid nodules (^39^). In this research, as reflected by the results of US-FNAB, CT and *BRAF^V600E^* gene detections, the PTC group displayed augmented positive detection rates of the three test modalities relative to the NG group. This is supported by an existing report that ultrasonographic characteristics have been detected many times for discriminating benign from malignant thyroid nodules, whereas US-FNAB is viewed as the existing standard for precise diagnosis of thyroid nodules (^40^). Positron emission tomography/CT-positive thyroid nodules usually have elevated malignancy rates, warranting further investigation to elucidate the characteristics of these nodules (^41^). Moreover, Du and cols. have confirmed that *BRAF^V600E^* mutation rates are elevated in the papillary thyroid microcarcinoma compared with benign lesions like NG, Hashimoto’s thyroiditis with fibrosis and calcification, and calcification (^42^). Taken together, it is plausible to conclude that PTC patients exhibited higher positive rates of US-FNAB, CT, and *BRAF^V600E^* gene mutation than those with benign thyroid nodules.

Furthermore, our study demonstrated that the positive detection rates of US-FNAB and *BRAF^V600E^* were higher in patients at TNM stages II-III than those at stage I. The detection rates of positive *BRAF^V600E^* and CT in patients with lymphatic metastasis were higher than those without lymphatic metastasis. There is research revealing that US-FNAB is regarded as the preferred method for evaluating thyroid nodules and lymph nodes in patients with suspected thyroid cancer (^43^). In contrast to ultrasonography, CT is not impeded by gas and bone, allowing for superior visualization of central-level lymph node metastasis in PTC (^44^). Additionally, *BRAF^V600E^* mutation is associated with adverse clinicopathological outcomes in PTC, including lymphatic metastasis, advanced TNM stage, and patient mortality (^45^,^46^). Also, some experts indicate that *BRAF^V600E^* mutations can markedly elevate the likelihood of central lymph node metastasis in patients with PTC (^46^,^47^). Combined with our findings, US-FNAB and *BRAF^V600E^* gene detection results were interrelated to the clinical TNM stage of PTC patients, whereas CT and *BRAF^V600E^* gene detection results were linked to lymphatic metastasis. Furthermore, our results demonstrated that the AUC of the combination of US-FNAB, CT and *BRAF^V600E^* gene detections was obviously higher than those of them alone. Similarly, US-FNAB combined with *BRAF^V600E^* is suggested to intensify the diagnostic accuracy of macro-calcified thyroid nodules, with a markedly higher sensitivity (^13^). The supplementary benefit of CT combined with ultrasound for evaluating lymph node metastasis in thyroid cancer has been examined in existing studies, and their combination demonstrated enhanced sensitivity and diagnostic value relative to ultrasound or CT used independently (^48^,^49^). Zhang and cols. have revealed that enhanced CT, when combined with *BRAF^V600E^* gene detection, enhances the diagnostic accuracy for PTC, demonstrating superior clinical indicators and safety compared to fine-needle aspiration cytology (^50^). Of note, this study for the first time explored the diagnostic value of the three detection methods and their combined applications in PTC. We concluded that all of the US-FNAB, CT and *BRAF^V600E^* gene tests had high diagnostic value for PTC, and the diagnostic value of their combined detection surpassed that of the single detection.

In summary, this study used US-FNAB, CT and *BRAF^V600E^* gene tests to explore the biological conditions of thyroid cells and the status of BRAF gene, plotted ROC curves to analyze the diagnostic role of the three test methods for PTC, and analyzed the relationship of them with the pathohistological features of the PTC patients. The study provides reasonable and effective guidance for the preoperative diagnosis and condition evaluation of PTC patients. However, US-FNAB takes fewer cells and some of the cells are easy to be destroyed, highlighting higher technical requirements for the puncture doctor and the pathologist to read the slides during the actual operation. Beyond that, the influence of testing funds and patients’ wishes led to the limited sample size of the study. Furthermore, we will further carry out multicenter studies to expand the sample size, strictly control the technical variables, strengthen the professionalism of clinical pathologists and clinical operators, and reduce the interference of subjective and objective reasons on the research results, thereby enabling a more comprehensive and in-depth evaluation of the validity and reliability of these biochemical markers. Moreover, we will further investigate the influence of the pathologic and histologic characteristics of PTC patients on the outcomes of US-FNAB detection, CT detection and *BRAF^V600E^* gene mutation test, as well as the interaction between the outcomes of the three tests.

**Ethics approval and consent to participate:** this study was reviewed and approved by the Academic Ethics Committee of Hunan Provincial People’s Hospital, The First Affiliated Hospital of Hunan Normal University (2019-1223), and was in line with the Declaration of Helsinki and the Enhancing the Quality and Transparency Of health Research network guidelines. All subjects were informed of the purpose of the study and signed the informed consent forms.

## Data Availability

the data that support the findings of this study are available from the corresponding author upon reasonable request.
